# Relationship between Lower Limb Muscle Activation Characteristics and Running Economy in Recreational Runners

**DOI:** 10.5114/jhk/203469

**Published:** 2025-09-23

**Authors:** Shouxin Jiang, Christophe A. Hautier, Shiqin Chen, Yue Shi, Qingshan Zhang, Fei Li

**Affiliations:** 1 School of Athletic Performance, Shanghai University of Sport, Shanghai, China.; 2 Laboratoire Interuniversitaire de Biologie de la Motricité, Université de Lyon, Villeurbanne Cedex, France.; 3 Shanghai Key Lab of Human Performance, Shanghai University of Sport, Shanghai, China.

**Keywords:** sEMG, muscle activation, running economy, co-activation, SnPM

## Abstract

The aim of this study was to assess the correlation between lower limb muscle activation, co-activation characteristics, and running economy (RE). Twenty-nine male recreational runners participated in two sessions: during the first session, body composition and RE at 10 km•h^−1^ were measured, while during the second session, we assessed muscle activation using sEMG for rectus femoris (RF), vastus lateralis (VL), vastus medialis (VM), biceps femoris (BF), gluteus maximus (GM), tibialis anterior (TA), gastrocnemius lateralis (LG), gastrocnemius medialis (MG), and soleus (SOL) muscles. Additionally, seven pairs of agonist-antagonist co-activation ratios were calculated. Pearson's and statistical non-parameters mapping (SnPM) correlation analyses were used to evaluate the muscular activity and RE, respectively. Pearson’s analysis showed that GM, VM, VL, LG, MG, and SOL activation during the stance phase was negatively correlated with RE (r = −0.499 to −0.592, p < 0.05), whereas RF and VL activation during the initial swing phase was positively correlated with RE (r = 0.522, r = 0.527; p < 0.05). Regarding muscle co-activation, the SOL-TA in the propulsion, the BF-VL, and GM-RF during the initial swing phase were negatively correlated with RE (r = −0.506 to −0.634, p < 0.05). In contrast, the RF-BF in the terminal swing phase was positively correlated with RE (r = 0.578, p < 0.05). Unfortunately, SnPM analysis did not show significant results. In conclusion, greater lower limb muscle activation during the stance phase is associated with better RE. During the swing phase, the co-activation of the high activation of the posterior thigh muscles (GM, BF) and the lower activation of the anterior thigh muscles (quadriceps) is associated with better RE.

## Introduction

Running Economy (RE) is a key determinant of endurance running performance and is defined as the amount of oxygen consumed at a steady state while running at a specified sub-maximal speed. Better RE indicates a more efficient use of oxygen, which is essential for sustained performance in endurance events (Fletcher and MacIntosh, 2017). VO_2max_ is often thought to be the main factor influencing endurance levels, but as research has progressed, it has been found that runners with high levels of RE perform better on endurance runs than runners with similar levels of VO_2max_ (Foster and Lucia, 2007). Therefore, it is essential to investigate the factors influencing RE to enhance endurance running performance.

One study suggests that approximately 80% of the net metabolic cost of running is attributed to muscle activation and recruitment, which are necessary for generating the force required to support body weight and propel the runner forward (Arellano and Kram, 2014). Therefore, muscle activation, especially in the lower limbs, is considered an important factor influencing RE. However, only a limited number of studies have explored the relationships among these factors, and the research results are rather inconsistent. In terms of muscle activation, in several studies (Bohm et al., 2021; Tam et al., 2019), it was found that higher activation of the knee and ankle muscles was associated with better RE during running. From a biomechanical perspective, during running, the lower limb muscles play a crucial role in maintaining stability and providing propulsion. For example, the rectus femoris (RF) and the medial head of the gastrocnemius (GAS) are important bi-articular muscles. Activating these two muscles during the running stance phase can optimize the force distribution across multiple joints, thereby enhancing joint stability and efficiently converting the elastic energy stored in the tendons into propulsion (Tam et al., 2019). Secondly, previous studies have suggested that greater pre-activation of the leg extensors increases the sensitivity of muscle spindles by enhancing alpha-gamma co-activation and the stretch reflex, which may increase tendon stiffness and thus enhance RE (Santos-Concejero et al., 2014). However, higher muscle activation is also generally considered detrimental to RE (Carson et al., 2024; Monte et al., 2023). From a physiological perspective, higher muscle activation reflects more active cross-bridges and ion pumping, and thus higher energy costs (Fletcher and MacIntosh, 2017). Monte et al. (2023) demonstrated that as muscle activation increases, additional motor units are recruited to generate force, resulting in an increase in oxygen uptake. This conflicting finding may be related to the elasticity of the runner's muscle structure (extracellular matrix and muscle fiber itself), training experience, and test speed. In fact, muscle activation varies in different phases of the gait cycle, and it is necessary to further divide the gait cycle to explore the relationship between muscle activation and RE. However, existing studies simply divide it into the stance phase and the swing phase.

Meanwhile, agonist-antagonist co-activation refers to the simultaneous activation of muscle groups with opposing actions during a specific movement, which is essential for achieving limb coordination in human movement (Latash, 2018). Heise et al. (2008) showed that muscle co-activation can reduce metabolic costs by increasing lower limb joint stiffness and effectively utilizing elastic energy. However, several studies have found that increased co-activation of lower limb muscle may lead to increased metabolic costs (Lemineur et al., 2024; Moore et al., 2014; Tam et al., 2017). It is pointed out that the purpose of co-activation is to enhance joint stability and balance the pressure distribution on the joint surface to maintain the function of soft tissues. Excessive co-activation beyond the level required for joint stability may disrupt the efficiency of the stretch-shortening cycle (SSC) and lead to an increase in metabolic costs (Moore et al., 2014). These conflicting results may reflect the differences in the methods used to determine the co-activation time and muscle pairs. Existing studies lack a detailed exploration of the co-activation patterns of the main antagonist muscle pairs around the joints and their relationship with RE through the movement of the lower limb hip, knee, and ankle joints in different stages of the gait cycle.

Unfortunately, nearly all prior studies have relied on the conventional approach of using discrete variables derived from entire movement trajectories (e.g., mean EMG amplitude and mean activation time), which are subsequently correlated with RE. While this approach simplifies the complexity of statistical analysis, it tends to ignore the temporal continuity of the data. For this reason, we introduced Statistical Parametric Mapping (SPM), which is capable of assessing the statistical significance of the entire motion profile (Pataky, 2010). SPM is based on random field theory (Robinson et al., 2015) and calculates statistical differences between data by computing the portions of contiguous datasets exceeding thresholds (Pataky, 2012). Thus, by utilizing time-series analysis, we can identify time ranges of positive/negative correlations, thereby gaining complementary information to understand the relationship between muscle activity and RE throughout the gait cycle.

The purpose of this study was to assess the relationship between lower limb muscle activation levels and agonist-antagonist co-activation and RE during the running gait cycle at low speeds in male recreational runners. The second aim was to investigate the correlation between RE and muscle RMS time-series data based on RE under SPM. We hypothesized that 1) during the propulsion phase, high activation of lower limb muscles, particularly the calf triceps, would be associated with better RE; 2) during the swing phase, excessive lower limb muscle activation would be associated with poorer RE; 3) during the running cycle, lower limb muscle co-activation would be associated with RE. Focusing on discrete point analyses and continuous variable analyses can compensate for the limitations of a single approach, thus revealing the complex relationship between muscle activation and RE at multiple levels.

## Methods

### Participants

A total of twenty-nine male recreational runners from the university running club volunteered to participate in the study. The sample size calculation was performed using G*power 3.1 software. We used the setting two-sided test, α err = 0.05, Power (1-β err = 0.80) to satisfy the power requirements of the bivariate normal model (Faul et al., 2009). Table 1 shows the basic demographics of the participants. Participants had to meet the following criteria: (1) run at least three times per week, (2) run a minimum of 30 km per week for 3 months, (3) get used to a rearfoot strike pattern. The study was approved by the ethics committee of the Shanghai University of Sport, Shanghai, China (approval code: 102772023RT107; approval date: 13 October 2023). All participants gave written informed consent before participating in the study.

**Table 1:** The basic demographics of study participants.

Variable	Mean ± SD
Age (years)	21.00 ± 1.00
Body height (cm)	179.53 ± 5.64
Body mass (kg)	72.48 ± 10.01
BMI (kg•cm^−2^)	22.39 ± 1.90
FFM (kg)	61.24 ± 7.29
FM (kg)	11.23 ± 3.91
VO_2max_ (ml•kg^−1^•min^−1^)	54.58 ± 4.61
HR (bpm•min^−1^)	156.0 ± 12.30
RER	0.90 ± 0.06
RE (ml•kg^−1^•min^−1^)	40.79 ± 2.19

Values represent mean ± SD. BMI: body mass index; FFM: fat-free mass; FM: fat mass; VO_2max:_ maximum oxygen uptake; RER: respiratory exchange ratio; HR: heart rate; RE: running economy

### Design and Procedures

This study had a cross-sectional design. In order to prevent the possible influence of running biomechanical testing equipment (reflective markers and Surface Electromyography electrodes) on the RE test, each participant completed two separate testing sessions in the laboratory with at least 48 h of rest between tests. Before each session, participants were instructed to get at least 8 h of sleep, avoid strenuous exercise for 24 h, and be without muscle soreness and fatigue. Two weeks prior to the test, runners were asked to enter the lab twice a week for 30 min of practice runs at the RE test speed to fully acclimatize to the RE test conditions. During the first testing session, participants' body composition and RE at 10 km•h^−1^ were measured; during the second testing session, participants' biomechanical indices were assessed, including the activation of nine muscles and the co-activation ratios of seven pairs of agonist-antagonist muscles at a speed of 10 km•h^−1^.

### Body Composition Test

Standing height was measured with a wall-mounted measuring device (Butterfly, Shanghai, China). Body mass, fat mass, and fat-free mass were measured using a bioimpedance analyzer (X-scan Plus II; Jawon, South Korea) and the body mass index (BMI) was calculated according to the formula: BMI = body mass (kg) / body height (m^2^).

### Running Economy Test

As participants were accustomed to treadmill running, all subsequent running tests were completed on a motorized treadmill (Bertec, FIT, USA). They began with a 4-min warm-up at a pace of 8 km•h^−1^. After a 5-min rest interval, they ran at 10 km•h^−1^ for 4 min to determine RE. RE was determined by averaging the oxygen uptake (ml•kg^−1^•min^−1^) recorded during the final minute of the steady-state running phase. Based on the pre-test questionnaire, participants reported that their daily training typically involved running at a speed of 10 km•h^−1^. Research suggests that runners achieve optimal energy efficiency when running at their habitual pace (Jones and Carter, 2000). Moreover, previous studies investigating recreational runners frequently utilized 10 km•h^−1^ as a standard speed for assessing RE (Chen et al., 2024; Tam et al., 2019). A portable metabolic analyzer (K5, Cosmed, Rome, Italy) and a heart rate monitor belt (Garmin, Olathe, KS) were used to continuously measure oxygen uptake and the heart rate (HR) throughout the test.

### Running Biomechanics and Surface Electromyography (sEMG) Test

**Figure 1:**
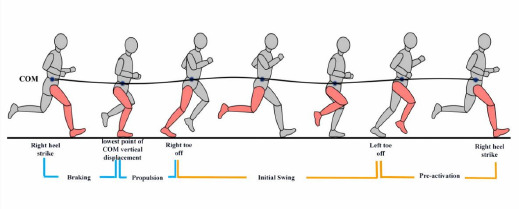
Schematic diagram illustrating phases of the running gait cycle. COM: Center of Mass. These phases include: Braking phase: from the right heel strike to the lowest point of COM vertical displacement. Propulsion phase: from the lowest point of COM vertical displacement to the right toe-off. Initial swing phase: from the right toe-off to the left toe-off. Pre-activation phase (terminal swing phase): from the left toe-off to the next right heel strike

Prior to the running test, participants were required to wear identical running shoes and tight pants. A total of 36 reflective markers were installed to identify the hip, knee and ankle joints (Xia et al., 2017) (Figure 1). An 8-camera, 3-dimensional (3D) motion capture system (Vicon T40; Oxford Metrics, Oxford, United Kingdom) was used to record the position of 36 reflective markers (100 Hz) to accurately obtain vertical displacements of the center of mass (COM) (calculated by the body segmental analysis technique (Gard et al., 2004). Vertical ground reaction force (vGRF) data were collected by means of two Kistler 3D force plates (9287 B, 90 cm × 60 cm × 10 cm, Kistler Corporation) embedded in the treadmill belt sampled at 1000 Hz. A wireless surface EMG (Noraxon Ultium EMG, 2000 Hz, United States) system was used to collect the muscular activities of the lower limb, including nine muscles of the right leg (gluteus maximus [GM], rectus femoris [RF], vastus lateralis [VL], vastus medialis [VM], biceps femoris [BF], tibialis anterior [TA], gastrocnemius lateralis [LG], gastrocnemius medialis [MG], and soleus [SOL]). Before testing, body hair was removed from the electrode placement sites using a razor and wiped with alcohol. Following skin preparation, surface electrodes were positioned on the muscle bellies of the target muscles, aligned with the direction of the muscle fibers in accordance with the SENIAM guidelines (Hermens et al., 2000). To ensure accurate placement and signal quality, participants were instructed to activate their lower limb muscles and perform a series of in-place jumps. The electrode positions and the integrity of the EMG signals were verified using a computer interface, allowing for the detection and elimination of noise interference and baseline drift. Once confirmed, the electrodes were securely fastened with elastic straps to prevent detachment during data collection. The reliability of our EMG data acquisition was validated by an intraclass correlation coefficient (ICC) assessment indicating a high degree of consistency (ICC = 0.92, 95%CI: [0.86, 0.96]). After a 4-min warm-up at 8 km•h^−1^, participants carried out maximum force contractions for each muscle in standard postures. This allowed for the accurate acquisition of the EMG activity of each muscle during maximum voluntary contraction (MVC), further determining its peak amplitude for the normalization of EMG data during subsequent running. The specific postures for each muscle were as follows (Burden, 2010): for the GM, lie prone and lift the thigh by approximately 20°; for the BF, lie prone and flex the knee between 20° and 30°; for the quadriceps (RF, VM, VL), sit and extend one knee with the knee flexion angle between 70° and 90°; for the TA, stand and dorsiflex the toes; for the GAS (LG, MG), sit with straight knees and perform unilateral plantar flexion at the ankle joint at 90°; for the SOL, sit and perform ankle extension and plantar flexion.

Participants then ran at 10 km•h^−1^ for 4 min. Data were collected for 15 s for each participant in the last minute (at least 10 consecutive steps) (Folland et al., 2017). A digital-to-analog converter was used to synchronize motion capture and sEMG data.

### Data Processing

The vGRF and COM were processed by Visual 3-dimensional (3D) gait analysis software (v3, C-Motion, Inc., Germantown, MD, United States). The vGRF and COM raw data were filtered using a Butterworth second-order bidirectional low-pass filter with a cut-off frequency of 50 Hz (Zhou et al., 2021). A vertical force signal of 50 N (~7% BW) was utilized to identify the initial foot-strike and toe-off (Kyröläinen et al., 2001). The raw EMG signals were processed using Matlab R2023a software. A fourth-order Butterworth band-pass filter with a 20–400-Hz cut-off frequency was applied to eliminate motion artifacts (Donath et al., 2015). The EMG signals were then normalized by selecting the peak amplitude of each muscle during the MVC test (Besomi et al., 2020). The root mean squared (RMS) value of the EMG signal was evaluated in a 50-ms moving window (Tam et al., 2019). The average RMS of the running gait cycle was calculated for ten consecutive steps. The running gait cycle was divided into four key phases by vGRF and vertical displacement of the body's COM (Figure 1) (Dugan and Bhat, 2005; Kyröläinen et al., 2001).

During running, the hip, knee, and ankle joints function in a coordinated manner. The level of co-activation between the muscles surrounding these joints may influence joint stability and, consequently, RE. Based on previous studies (Moore et al., 2014; Tam et al., 2017), we selected seven pairs of major antagonist muscles around these three joints and calculated their co-activation rates: RF and GM, RF and BF, VL and BF, VM and BF, MG and TA, LG and TA, and SOL and TA. The co-activation ratio was taken as $\frac{RMS{\;}_{Agonist}}{RMS_{Antagonis}}$ calculation (Tam et al., 2019). Agonist-antagonist ratios greater than 1.0 indicate more activation of the agonist muscle. Ratios less than 1.0 indicate more activation of the antagonist muscle. Ratios equal to 1.0 indicate equal activation of both muscles. The average values of all the aforementioned variables of 10 consecutive strides were used for the following process (Zverev, 2006).

### Statistical Analyses

First, the Shapiro-Wilk test was used to assess the normality of all discrete data. If the data conformed to the normal distribution, Pearson's product-moment correlation analysis was then adopted to analyze the relationships between the activation of lower limb muscles, the co-activation ratios at different stages of the running cycle, and RE. The magnitude of the correlation coefficient (r) was interpreted using the following criteria: small (0.1–0.3), moderate (0.3–0.5), large (0.5–0.7), very large (0.7–0.9), and extremely large (0.9–1.0) (Hopkins et al., 2009). All reported *p* values were adjusted using the Benjamini-Hochberg procedure, in order to correct false-discovery rates arising from statistical multiple comparisons (Benjamini and Hochberg, 1995). The coefficient of variation (CV) of participants’ muscle RMS was calculated to assess inter-individual differences in muscle activation, and the muscle activation of our participants exhibited a high coefficient of variation (CV ≥ 33.37%). We also calculated the coefficient of determination (r^2^), which is the square of the correlation coefficient (r). The r^2^ value represents the proportion of the variance in one variable that can be explained by the other variable in the linear relationship. In addition, we computed the 95% confidence interval (CI) for the correlation coefficient. The confidence interval provides a range within which the true population correlation coefficient is likely to be. A narrower confidence interval indicates a more precise estimate of the relationship. Statistical analyses were performed using SPSS (version 26.0; IBM Corp. Armonk, NY, United States) and the statistical programming language R (www.r-project.org). The threshold for statistical significance was set at *p* < 0.05. All the data were expressed as mean ± SD.

After time-normalizing the RMS of the nine lower limb muscles, to verify the robustness of the analysis results, this study employed both parametric and non-parametric regression methods to analyze the data. The parametric test assumes that the residuals are independently and identically distributed and follow a normal distribution, whereas the non-parametric method does not rely on such distributional assumptions. Since our data did not meet the normality assumption in the residual analysis, we reported the results of the non-parametric analysis. Non-parametric regression was performed using SnPM from the MATLAB package (https://spm1d.org) (Pataky, 2010) to determine the correlation between RE and lower limb muscle activation trajectories. A permutation test was used with 10,000 permutations to determine a significance threshold for an alpha error of 0.05, and a Pearson's r value was calculated for each trajectory node. The magnitude of the r value was interpreted using the same criteria as for the discrete analyses.

## Results

As shown in Table 2, during the braking phase, the activation level of GM was moderately negatively correlated with RE (r = −0.499, *p* = 0.045, r^2^ = 0.249, 95%CI: [−0.739, −0.147]). Furthermore, during the propulsion phase, the degree of activation of the VL, VM, LG, MG, and SOL muscles was moderate to highly negatively correlated with RE (r = −0.500 to −0.592, *p* < 0.05, r^2^ = 0.250 to 0.350, 95%CI: [−0.793 to −0.739, −0.273 to −0.148]). During the braking phase, there was no significant correlation between the muscle co-activation ratio and RE. In contrast, during the propulsion phase, there was a significant negative correlation between the SOL-TA co-activation ratio and RE (r = −0.506, *p* = 0.041, r^2^ = 0.256, 95%CI: [−0.743, −0.156]).

**Table 2:** Correlation between muscle RMS, the muscle co-activation ratio and RE during the stance phase at 10 km•h^−1^.

Variable	RE at 10 km•h^−1^						
	Mean ± SD	CV	r	r^2^	95%CI		*p* value
**Muscle RMS (Braking phase)**						
RF (%)	10.94 ± 8.02	73.31	−0.280	0.078	−0.597	0.112	0.307
VL (%)	9.96 ± 7.83	78.61	−0.364	0.132	−0.654	0.018	0.152
VM (%)	12.14 ± 9.56	78.75	−0.416	0.173	−0.687	−0.043	0.107
BF (%)	8.31 ± 6.05	72.80	−0.060	0.004	−0.430	0.327	0.856
GM (%)	13.17 ± 7.84	59.53	−0.499*	0.249	−0.739	−0.147	**0.045**
TA (%)	10.21 ± 5.29	51.81	−0.361	0.130	−0.651	0.022	0.152
LG (%)	8.33 ± 5.59	67.11	−0.274	0.075	−0.592	0.119	0.313
MG (%)	8.80 ± 6.67	75.80	−0.223	0.050	−0.556	0.171	0.430
SOL (%)	9.50 ± 7.10	74.74	−0.378	0.143	−0.663	0.002	0.138
**Muscle RMS (Propulsion phase)**						
RF (%)	8.31 ± 5.31	63.90	−0.336	0.113	−0.635	0.050	0.184
VL (%)	7.72 ± 5.92	76.68	−0.500*	0.250	−0.739	−0.148	**0.045**
VM (%)	7.81 ± 5.69	72.86	−0.505*	0.255	−0.742	−0.154	**0.045**
BF (%)	8.84 ± 6.04	68.33	−0.148	0.022	−0.500	0.246	0.651
GM (%)	9.51 ± 5.86	61.62	−0.422	0.178	−0.691	−0.049	0.107
TA (%)	6.81 ± 4.28	62.85	−0.086	0.007	−0.451	0.304	0.856
LG (%)	10.91 ± 7.02	64.34	−0.569*	0.324	−0.780	−0.240	**0.045**
MG (%)	12.08 ± 7.67	63.49	−0.502*	0.252	−0.740	−0.150	**0.045**
SOL (%)	12.07 ± 8.04	66.61	−0.592*	0.350	−0.793	−0.273	**0.045**
**Muscle co-activation (Braking phase)**						
RF-GM	0.87 ± 0.50	57.47	0.392	0.154	0.014	0.672	0.188
BF-RF	1.88 ± 2.69	143.09	0.028	0.001	−0.355	0.404	0.888
BF-VL	3.02 ± 5.81	192.38	0.136	0.018	−0.257	0.491	0.647
BF-VM	2.26 ± 3.10	137.17	0.184	0.034	−0.211	0.527	0.503
TA-MG	5.62 ± 8.76	155.87	−0.037	0.001	−0.412	0.348	0.884
TA-LG	3.92 ± 4.80	122.45	−0.036	0.001	−0.410	0.349	0.884
TA-SOL	3.06 ± 3.08	100.65	0.228	0.052	−0.166	0.560	0.429
**Muscle co-activation (Propulsion phase)**						
GM-RF	2.12 ± 2.65	125.00	0.052	0.003	−0.335	0.423	0.873
RF-BF	1.95 ± 2.49	127.69	0.115	0.013	−0.278	0.474	0.687
VL-BF	1.40 ± 1.78	127.14	−0.219	0.048	−0.553	0.176	0.429
VM-BF	1.33 ± 1.24	93.23	−0.278	0.077	−0.595	0.114	0.389
MG-TA	3.04 ± 2.89	95.07	−0.253	0.064	−0.578	0.140	0.389
LG-TA	2.60 ± 2.68	103.08	−0.259	0.067	−0.582	0.134	0.389
SOL-TA	2.90 ± 2.50	86.21	−0.506*	0.256	−0.743	−0.156	**0.041**

Values represent mean ± SD. * Significant correlation (* p < 0.05, ** p < 0.001). Significant effects are reported in bold font. CV: coefficient of variation; RMS: root mean square amplitude; RF: rectus femoris; VL: vastus lateralis; VM: vastus medialis; BF: biceps femoris; GM: gluteus maximus; TA: tibialis anterior; LG: gastrocnemius lateralis; MG: gastrocnemius medialis; SOL: soleus; GM-RF: gluteus maximus-rectus femoris; RF-BF: rectus femoris-biceps femoris; VL-BF: vastus lateralis-biceps femoris; VM-BF: vastus medialis-biceps femoris; LG-TA: gastrocnemius lateralis-tibialis anterior; MG-TA: gastrocnemius medialis-tibialis anterior; SOL-TA: soleus-tibialis anterior; RF-GM: rectus femoris-gluteus maximus; BF-RF: biceps femoris-rectus femoris; BF-VL: biceps femoris-vastus lateralis; BF-VM: biceps femoris-vastus medialis; TA-LG: tibialis anterior-gastrocnemius lateralis; TA-MG: tibialis anterior-gastrocnemius medialis; TA-SOL: tibialis anterior-soleus

As shown in Table 3, the activation level of the RF and VL muscles showed a large positive correlation with RE during the initial swing phase (r = 0.522; r = 0.527, *p* = 0.045, r^2^ = 0.272; r^2^ = 0.278, 95%CI: [0.177, 0.753]; 95%CI: [0.184, 0.756]). However, no significant correlation was found between the degree of muscle activation and RE in the pre-activation phase. The co-activation ratio of BF-VL showed a large negative correlation with RE (r = −0.634, *p* < 0.001, r^2^ = 0.402, 95%CI: [−0.817, −0.335]) in the initial swing phase. We observed that during the pre-activation phase, the co-activation ratio of GM-RF muscles had a large negative correlation with RE (r = −0.604, *p* = 0.014, r^2^ = 0.365, 95%CI: [−0.800, −0.291]). Conversely, the co-activation ratio between the RF and BF muscles showed a large positive correlation with RE (r = 0.578; *p* = 0.014, r^2^ = 0.334, 95%CI: [0.253, 0.785]).

**Figure 2:**
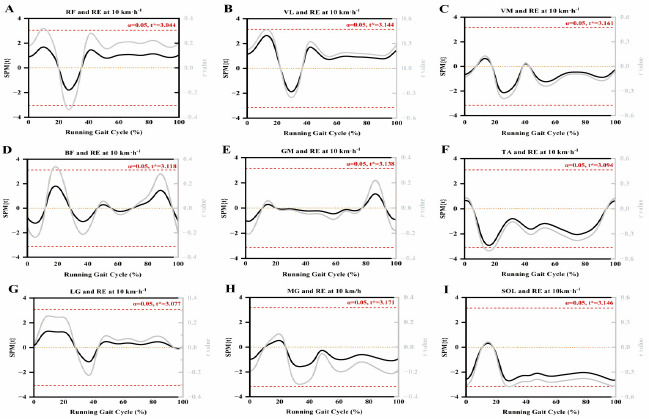
SnPM curves of lower limb muscle RMS and running economy during the running gait cycle. Red dotted line: the critical threshold calculated by SnPM; RF: rectus femoris; VL: vastus lateralis; VM: vastus medialis; BF: biceps femoris; GM: gluteus maximus; TA: tibialis anterior; LG: gastrocnemius lateralis; MG: gastrocnemius medialis; SOL: soleus

As shown in Figure 2, no significant correlation was found between lower limb muscle activity and RE during the gait cycle after conducting the SnPM analysis on the data. Some of our findings showed a trend of significant correlation. Figure 2 shows that TA and SOL activation were almost correlated with RE during the support phase and that the trend in r values was similar to that of traditional discrete analyses.

**Table 3:** Correlation between muscle RMS, the muscle co-activation ratio and RE during the swing phase at 10 km•h^−1^.

Variable	RE at 10 km•h^−1^						
	Mean ± SD	CV	r	r^2^	95%CI		*p* value
**Muscle RMS (Initial swing phase)**						
RF (%)	5.95 ± 4.78	80.34	0.522*	0.272	0.177	0.753	**0.045**
VL (%)	4.18 ± 4.82	115.31	0.527*	0.278	0.184	0.756	**0.045**
VM (%)	4.32 ± 4.41	102.08	0.452	0.204	0.087	0.710	0.085
BF (%)	7.24 ± 4.10	56.63	0.217	0.047	−0.178	0.552	0.430
GM (%)	8.56 ± 4.27	49.88	0.047	0.002	−0.339	0.419	0.856
TA (%)	10.16 ± 3.39	33.37	0.037	0.001	−0.348	0.411	0.856
LG (%)	4.73 ± 4.43	93.66	0.332	0.110	−0.055	0.632	0.186
MG (%)	4.80 ± 4.98	103.75	0.399	0.159	0.023	0.677	0.110
SOL (%)	5.25 ± 5.08	96.76	0.338	0.114	−0.048	0.637	0.184
**Muscle RMS (Pre-activation phase)**						
RF (%)	5.46 ± 4.94	90.48	0.220	0.048	−0.175	0.553	0.430
VL (%)	5.07 ± 4.68	92.31	0.044	0.002	−0.341	0.417	0.856
VM (%)	5.86 ± 5.43	92.66	−0.041	0.002	−0.415	0.344	0.856
BF (%)	12.56 ± 7.77	61.86	−0.448	0.201	−0.708	−0.082	0.085
GM (%)	9.66 ± 5.57	57.66	−0.055	0.003	−0.426	0.332	0.856
TA (%)	13.18 ± 5.79	43.93	−0.399	0.159	−0.677	−0.023	0.110
LG (%)	4.52 ± 5.79	128.10	0.217	0.047	−0.178	0.551	0.856
MG (%)	4.57 ± 5.54	121.23	0.206	0.042	−0.188	0.544	0.439
SOL (%)	4.77 ± 4.92	103.14	0.120	0.014	−0.273	0.478	0.731
**Muscle co-activation (Initial swing phase)**						
RF-GM	0.81 ± 0.67	82.72	0.360	0.130	−0.023	0.651	0.228
BF-RF	2.38 ± 2.17	91.18	−0.249	0.062	−0.575	0.144	0.389
BF-VL	4.92 ± 3.23	65.65	−0.634**	0.402	−0.817	−0.335	**0.000**
BF-VM	3.85 ± 2.58	67.01	−0.371	0.138	−0.658	0.011	0.222
TA-MG	7.07 ± 6.48	91.65	−0.124	0.015	−0.482	0.268	0.670
TA-LG	6.44 ± 6.77	105.12	−0.081	0.007	−0.447	0.309	0.804
TA-SOL	4.68 ± 3.59	76.71	−0.060	0.004	−0.430	0.325	0.864
**Muscle co-activation (Pre-activation phase)**						
GM-RF	2.88 ± 1.55	53.82	−0.604*	0.365	−0.800	−0.291	**0.014**
RF-BF	1.22 ± 2.43	199.18	0.578*	0.334	0.253	0.785	**0.014**
VL-BF	0.78 ± 1.02	130.77	0.143	0.020	−0.251	0.496	0.642
VM-BF	0.80 ± 0.83	103.75	0.396	0.157	0.019	0.675	0.188
TA-MG	11.89 ± 17.27	145.25	−0.249	0.062	−0.574	0.145	0.389
TA-LG	9.23 ± 7.51	81.37	−0.289	0.084	−0.603	0.102	0.389
TA-SOL	6.30 ± 4.95	78.57	−0.211	0.045	−0.547	0.184	0.429

Values represent mean ± SD. * Significant correlation (* p < 0.05, ** p < 0.001). Significant effects are reported in bold font. CV: coefficient of variation; RMS: root mean square amplitude; RF: rectus femoris; VL: vastus lateralis; VM: vastus medialis; BF: biceps femoris; GM: gluteus maximus; TA: tibialis anterior; LG: gastrocnemius lateralis; MG: gastrocnemius medialis; SOL: soleus; GM-RF: gluteus maximus-rectus femoris; RF-BF: rectus femoris-biceps femoris; VL-BF: vastus lateralis-biceps femoris; VM-BF: vastus medialis-biceps femoris; RF-GM: rectus femoris-gluteus maximus; BF-RF: biceps femoris-rectus femoris; BF-VL: biceps femoris-vastus lateralis; BF-VM: biceps femoris-vastus medialis; TA-LG: tibialis anterior-gastrocnemius lateralis; TA-MG: tibialis anterior-gastrocnemius medialis; TA-SOL: tibialis anterior-soleus

## Discussion

**Figure 3:**
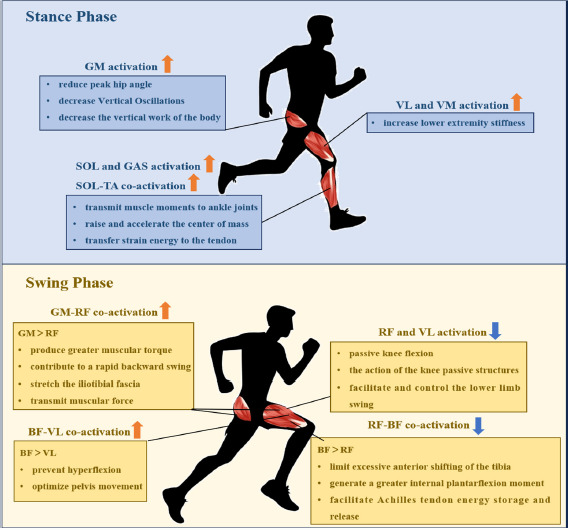
The potential summary mechanisms of lower extremity muscle activation regulating running economy. RF: rectus femoris; VL: vastus lateralis; VM: vastus medialis; BF: biceps femoris; GM: gluteus maximus; SOL: soleus; GAS: gastrocnemius; SOL-TA: soleus-tibialis anterior; GM-RF: gluteus maximus-rectus femoris; BF-VL: biceps femoris-vastus lateralis; RF-BF: rectus femoris-biceps femoris

The present study explored the relationship between lower limb muscle activation and RE during the running gait cycle. The potential mechanisms by which muscle activation in the lower limbs during running affects RE are shown in Figure 3.

The first significant finding of this study was the observed relationship between greater lower limb muscle activation and improved RE during the stance phase. The mechanism for the reduced oxygen cost associated with increased lower limb muscle activation during the stance phase may involve higher muscle activation absorbing landing impacts, optimizing joint stiffness and stability, thereby reducing Vertical Oscillations (VOSC) during the stance phase and maximizing the release and reuse of elastic potential energy. During the initial stance phase (i.e., braking phase), the lower limbs receive an external shock load, and the magnitude of the load could be 2–3 times the body weight, which requires the lower limb muscles to activate quickly to absorb and buffer the impact (Lieberman et al., 2010). As the lower extremity absorbs body weight, the hip flexes slightly, after which it begins to extend, and the GM is activated to control trunk flexion and decelerate the swing leg (Lieberman et al., 2006). It is unsurprising that our study indicated the negative correlation between GM activation and RE (r = −0.499, *p* = 0.045). It could be suggested that increased GM muscle activity during the braking phase helps to prevent hip hyperflexion (Kyröläinen et al., 2001), which may reduce the peak hip angle and decrease VOSC of the body's COM, decrease the vertical work of the body, and thus reduce oxygen uptake during running (Chen et al., 2024).

After the braking phase, the lower limb muscle works from eccentric to concentric, oscillating upward and shifting the forward body's center of gravity by generating the forward ground reaction force during the mid-to-late stance phase, thus the propulsion phase. During the propulsion phase, we found that VL, VM, LG, MG and SOL activation was moderately to highly negatively correlated with RE (r = −0.500 to −0.592, *p* < 0.05). The knee shifts from flexion to extension as the center of gravity moves anteriorly towards the knee (Heise et al., 2008; Tam et al., 2019). As lower limb muscle activation increases, lower limb stiffness during dynamic exercise increases (Tam et al., 2017), and appropriate lower limb stiffness allows the elastic energy stored in the tendon to be efficiently released and provide propulsive force, thus reducing the metabolic cost of muscle contraction (Brazier et al., 2019). Interestingly, we found that the effect size for the correlation between muscle activation and oxygen uptake in the lower limb during propulsion appeared to be larger in the distal lower limb. Our findings are supported by Tam et al. (2019). It is possible that due to the shift from dorsiflexion to plantarflexion of the ankle during the propulsion phase, the GAS, as a bi-articular muscle, plays a crucial role in transmitting muscle moments from the proximal joints to the ankle joints (Tam et al., 2019) and the SOL, as the largest muscle in the calf, is responsible for raising and accelerating the center of mass (Bohm et al., 2019). Moreover, a significant correlation exists between the co-activation ratio of the SOL-TA and RE during the propulsion phase (r = −0.506, *p* = 0.041). Compared to the TA, the active contraction of the SOL transfers more strain energy to the tendon, contributing to energy conservation (Bohm et al., 2019). In summary, higher lower limb muscle activation, particularly of the calf triceps, was associated with less oxygen uptake during the propulsion phase.

After the right toe-off, the body is thrust into the initial swing phase. During this phase, the lower limb muscle transmits from concentric to eccentric again. It suggests that the quadriceps work concentrically to swing the thigh forward, and the hamstring works eccentrically to decelerate the tibia to control the knee's range of motion (ROM) properly before the next foot contact (Chen et al., 2024; Dugan and Bhat, 2005). The present study found that RF and VL activation levels were positively correlated with RE (r = 0.522; r = 0.527, *p* < 0.045), suggesting that excessive activation may increase oxygen uptake. However, this finding contrasts with the results of Heise et al. (1996), who reported that RF activation during the early swing phase was associated with better RE. In addition, we found a large negative correlation between the co-activation ratio of BF-VL during the initial swing phase and RE (r = −0.634, *p* < 0.001). This finding is similar to that of Sundby and Gorelick (2014). They pointed out that a relatively higher activation intensity of hamstrings to quadriceps was associated with a lower running metabolic cost. This discrepancy in results may stem from the complex interplay of mechanical and neurological mechanisms. From a mechanical perspective, excessive activation of RF and VL may increase oxygen uptake while interfering with the elastic function of passive knee structures, such as tendons, thereby reducing energy efficiency (Bohm et al., 2019; Monte et al., 2020). Conversely, optimized activation of the hamstrings can improve energy utilization by limiting excessive forward tibial movement and enhancing hip joint stability (Folland et al., 2017). Secondly, during the initial swing phase, active flexion of the hip leads to passive flexion of the knee, with a subsequent increase in the passive moment of the knee (Heger et al., 2012). Knee passive structures appear to facilitate and control the lower limb swing by storing and releasing elastic potential energy (Heger et al., 2012). Due to the effect of the passive structure of the knee, the activation of the knee extensors (quadriceps) is reduced, thereby decreasing high and continuous traction (Zhang et al., 2024). This reduction may be beneficial in lowering knee joint pressure and minimizing energy expenditure in the lower limb joints. From a neurological perspective, insufficient neuromuscular coordination in recreational runners may lead to compensatory overactivation of RF and VL muscles during the swing phase, while the lack of optimized co-activation patterns further limits energy transfer efficiency (Clermont et al., 2019). These findings suggest that RE is influenced by the combined effects of dynamic mechanical regulation and neuromuscular coordination.

After the left toe-off, the pre-activation phase begins (the terminal swing phase), where the swinging limb prepares to contact the ground. At this point, hip flexion comes to an end, and hip extension initiates, driven by the concentric action of the GM and hamstrings (Dugan and Bhat, 2005). A key role of the pre-activation phase is to prepare for the upcoming touchdown for which lower limb proximal muscle co-activation optimized oxygen uptake after the initial foot contact. On the basis of the previous studies, co-activation of lower limb proximal muscles during the swing phase may reduce metabolic costs by optimizing neuromuscular control, assisting effective joint movement (especially hip extension and stabilization) (Yokozawa et al., 2007), and energy transfer (muscle conditioning) (Eng et al., 2015; Morin et al., 2015) compared to single muscles. First, considering that the GM-RF co-activation ratio was negatively associated with oxygen cost of transport (r = −0.604, *p* < 0.05), increased activation of GM produced greater muscular torque, contributing to a rapid backward swing of the leg before the foot contact (Yokozawa et al., 2007). When the GM is fully activated, it stretches the iliotibial fascia and transmits muscular force, storing elastic energy for the upcoming landing to conserve energy (Eng et al., 2015). Second, the RF-BF co-activation ratio was positively associated with oxygen transport cost (r = 0.578, *p* < 0.05), which is in line with Tam et al. (2017). Higher activation of the BF limits excessive anterior shifting of the tibia, contributing to leg retraction and a landing position closer to the body's side (Morin et al., 2015). This generates a greater internal plantarflexion moment to counteract dorsiflexion, facilitating Achilles tendon energy storage and release during the propulsion phase (Lieberman et al., 2010). Therefore, it can be observed that higher activation of the posterior thigh muscles (GM, BF) and lower activation of the anterior thigh muscles (quadriceps) during the swing phases were associated with better RE.

SPM, as a method of biomechanical analysis, enables the assessment of statistical significance across the entire motion curve (Pataky, 2010). Although the discrete analysis showed a significant correlation, the SnPM analysis did not show any significant effect. Nevertheless, we still found that the supporting phase TA and SOL activation were almost associated with RE, and the trend of r values was similar to the traditional discrete analysis. This result suggests that muscle activity may be more complex in its impact on RE than previously thought especially since the temporal scale has been normalized by the % of the gait cycle, which might decrease the susceptibility of significance. It could be suspected that RE could also be expressed by step or minute. Similarly to the previous literature (Del Vecchio et al., 2018), recreational runners in our study also showed high variability in muscle activation data (CV ≥ 33.37%), which suggests that there might be large individual differences in muscle activation among our study participants. However, this finding has important practical value. On the one hand, it highlights the pronounced heterogeneity in training backgrounds, technical proficiency, and physiological adaptation capacities among recreational runners, providing critical insights into the diversity of RE across populations. On the other hand, this finding underscores the necessity of personalized training approaches. High individual variability suggests that standardized training programs may not effectively meet the needs of all recreational runners. Running experience significantly influences muscle activation patterns, which in turn affect RE. Elite runners, through long-term training, optimize neuromuscular coordination, enabling precise regulation of joint stiffness and reduced metabolic cost. In contrast, recreational runners, due to a lack of training, exhibit high variability, resulting in a weaker or a non-significant relationship between muscle activation and RE. Additionally, the literature indicates that elite runners typically demonstrate highly consistent gait patterns, whereas the high variability observed in recreational runners reflects insufficient neuromuscular coordination (Clermont et al., 2019). Furthermore, discrete analysis may focus on specific features or measurement points in the data, which could amplify variability within the sample, especially when there are significant differences among individuals within the sample in physiological or biomechanical characteristics. SPM analysis, however, provides a holistic perspective by considering the spatial or temporal continuity of the data, which may fail to capture such differences (Robinson et al., 2015). Previous studies have rarely utilized the SPM analysis method to explore factors influencing RE. For instance, only Trowell et al. (2022) used SPM analysis, suggesting changes in biomechanics and muscle activity may not cause improvements in RE. Still, they did not investigate the correlation between them. Although the SnPM analysis did not reveal significant results in our study, we observed a trend in the r-values within SnPM, which closely mirrored our findings from the discrete analysis. In conclusion, SPM analysis has provided new insights into studying the correlation between muscle activity and RE. Future SPM analyses should consider larger sample sizes, utilize multivariate models, and incorporate multiple physiological and biomechanical variables to elucidate how they collectively impact RE.

The findings of this study highlight the importance of targeted strength training and biomechanical adaptations to improve RE. Specifically, the significant relationships between muscle activation patterns and RE suggest that runners should emphasize strengthening key muscle groups such as the hamstrings and calf triceps. Increased strength in these muscles can enhance joint stability, optimize lower limb stiffness, and facilitate efficient energy transfer, thereby reducing metabolic costs during running. Additionally, our findings underscore the need for coaches to focus on improving swing-phase biomechanics. Excessive anterior-posterior or left-right motion during the swing phase was associated with poorer RE (Chen et al., 2024), suggesting that runners should be trained to minimize unnecessary movements to reduce additional muscle activation and energy expenditure. Moreover, the observed high inter-individual variability in muscle activation patterns among recreational runners highlights the need for personalized training interventions. Practitioners should consider factors such as an athlete's training background, neuromuscular coordination, and running experience when designing training programs. Neuromuscular training and gait retraining exercises may be particularly beneficial for recreational runners to reduce variability and improve efficiency.

This study has certain limitations that should be acknowledged. First, the present findings suggest that muscle activation characteristics can serve as a reference index for explaining RE in male recreational runners. However, it is still to be determined whether these findings apply to high-level runners and female runners. Second, although the test speed (10 km•h^−1^) represented the habitual training pace for most recreational runners, it may not fully capture the variability in RE across different running speeds. Runners are known to exhibit differences in muscle activation patterns and biomechanical efficiency. By focusing on a single speed, this study may have overlooked speed-specific adaptations and their impact on RE, particularly in runners with varying levels of proficiency. Future studies should incorporate multiple running speeds to explore how RE and muscle activation vary across speeds, providing valuable insights into individualized training interventions and optimized performance. Additionally, habitual running styles should be thoroughly considered. Van der Meulen et al. (2024) demonstrated that recreational runners with a higher duty factor (DF) experience lower external and internal loading, which may influence muscle activation patterns and, consequently, energy expenditure efficiency. This finding suggests that future research should further explore the effects of different running habits on muscle activation patterns and RE in different populations. Finally, longitudinal studies focusing on the long-term effects of targeted interventions, such as strength training for specific muscle groups (e.g., hamstrings and calf muscles) and technical optimization, are recommended to evaluate their sustained impact on performance.

## Conclusions

In conclusion, the degree of lower limb muscle activation during different phases of running affected oxygen uptake and, consequently, RE. Higher lower limb muscle activation, particularly of the calf triceps, was associated with less oxygen uptake during the propulsion phase. During the swing phase, the synergistic effect of the high activation of the posterior thigh muscles (GM, BF) and the lower activation of the anterior thigh muscles (quadriceps) was associated with better RE. Unfortunately, we did not find a significant correlation using time-series (SnPM) analysis. These findings deepen our understanding of running biomechanical adaptations and provide valuable insights for training interventions. Future research should comprehensively consider running speed, runner population characteristics, and training intervention factors to more thoroughly investigate the relationship between muscle activation patterns and RE. Additionally, other key variables, such as energy expenditure, biomechanical variables, and psychological states, should be evaluated to provide more precise guidance for optimizing running performance.
